# Ginsenoside Rg3 Alleviates ox-LDL Induced Endothelial Dysfunction and Prevents Atherosclerosis in ApoE^−/−^ Mice by Regulating PPARγ/FAK Signaling Pathway

**DOI:** 10.3389/fphar.2020.00500

**Published:** 2020-04-22

**Authors:** Jianan Geng, Wenwen Fu, Xiaofeng Yu, Zeyuan Lu, Yanzhe Liu, Mingyang Sun, Ping Yu, Xin Li, Li Fu, Huali Xu, Dayun Sui

**Affiliations:** ^1^Department of Pharmacology, School of Pharmaceutical Sciences, Jilin University, Changchun, China; ^2^Institute of Traditional Chinese Medicine Innovation, Jilin Yatai Pharmaceutical Co., Ltd., Changchun, China; ^3^Institute of Dalian Fusheng Natural Medicine, Dalian Fusheng Pharmaceutical Co., Ltd., Dalian, China

**Keywords:** ginsenoside Rg3, atherosclerosis, monocyte adhesion, focal adhesion kinase, peroxisome proliferators-activated receptor γ, ApoE^−/−^ mice

## Abstract

The initiation of atherosclerosis (AS) induced by dyslipidemia is accompanied by endothelial dysfunction, including decreased healing ability and increased recruitment of monocytes. Studies showed ginsenoside Rg3 has potential to treat diseases associated with endothelial dysfunction which can protects against antineoplastic drugs induced cardiotoxicity by repairing endothelial function, while the effect and mechanism of Rg3 on dyslipidemia induced endothelial dysfunction and AS are not clear. Therefore, we investigated the effects of Rg3 on oxidized low-density lipoprotein (ox-LDL) induced human umbilical vein endothelial cells (HUVECs) dysfunction and high-fat diets (HFD) induced atherosclerosis in ApoE^−/−^ mice, as well as the mechanism. For *in vitro* assay, Rg3 enhanced healing of HUVECs and inhibited human monocytes (THP-1) adhesion to HUVECs disturbed by ox-LDL, down-regulated focal adhesion kinase (FAK)-mediated expression of vascular cell adhesion molecule 1 (VCAM-1) and intercellular adhesion molecule 1 (ICAM-1); restrained the FAK-mediated non-adherent dependent pathway containing matrix metalloproteinase (MMP)-2/9 expression, activation of nuclear factor-kappa B (NF-κB), high mRNA levels of monocyte chemotactic protein 1 (MCP-1) and interleukin 6 (IL-6), besides Rg3 up-regulated peroxisome proliferators-activated receptor γ (PPARγ) in ox-LDL-stimulated HUVECs. GW9662, the PPARγ-specific inhibitor, can repressed the effects of Rg3 on ox-LDL-stimulated HUVECs. For *in vivo* assay, Rg3 significantly reduced atherosclerotic pathological changes in ApoE^−/−^ mice fed with HFD, up-regulated PPARγ, and inhibited activation FAK in aorta, thus inhibited expression of VCAM-1, ICAM-1 in intima. We conclude that Rg3 may protect endothelial cells and inhibit atherosclerosis by up-regulating PPARγ *via* repressing FAK-mediated pathways, indicating that Rg3 have good potential in preventing dyslipidemia induced atherosclerosis.

## Introduction

Atherosclerosis (AS) is a chronic, low-grade inflammation-related disease. Dyslipidemia is one of the most common and important factors leading to atherosclerosis (Fava and Montagnana, 2018), notably, low-density lipoprotein (LDL) is a main contributor to AS, superabundant LDL can be oxidized into ox-LDL, which primary damages the endothelial barrier, interferes with endothelial healing function then emerges the subendothelial space (Goyal et al., 2012). Initiation of early AS induced by ox-LDL also accompanied with recruiting monocytes by endothelial cells derived ligands, mainly including vascular cell adhesion molecule 1 (VCAM-1) and intercellular adhesion molecule 1 (ICAM-1), the monocytes then enter into the subendothelial space and transform into foam cells, developing into atherosclerotic plaques (Takami et al., 1998; Tsai et al., 2016), thus it is important to protect function of endothelial cells against interference of exogenous factors in anti-atherosclerosis (Wu et al., 2017; Zhi et al., 2019). Furthermore, adhesion molecule expression requires a focal adhesion kinase (FAK) activation response, one study showed that inflammation promoted expression of ICAM-1 by up-regulating the FAK/protein kinase C delta signaling pathway in human fibroma cells, thus resulting in the infiltration of inflammatory cells to target organs (Huang et al., 2018). FAK is considered a major factor in promoting migration and invasion of tumor cells to normal tissue cells, and FAK up-regulated ICAM-1 expression that is involved in the cell motility of colorectal cancer (Taglia et al., 2007). Increasing evidence shows that FAK may be involved in the pathological processes of non-tumor-associated adhesion. Notably, FAK-mediated up-regulation of VCAM-1 is an important mechanism of endothelial injury by inflammatory infiltration induced by ox-LDL (Yurdagul et al., 2016). Chen et al. showed that the FAK-mediated nuclear factor kappa B (NF-κB) signaling pathway was involved in Ang II-accelerated atherosclerosis, characterized by the atherosclerotic lesion macrophage content and plaque instability increased (Chen et al., 2018), indicating that FAK may active non-adhesion dependent signals to accelerate AS.

Statins are considered as classic drugs to prevent atherosclerosis and concurrent cardiovascular disease by removing cholesterol, although there are high residual risks of insufficient LDL cholesterol reduction (Gragnano and Calabro, 2018), or liver enzyme activity increased after taking statins, resulting in an inability to tolerate full doses of statins for some patients (Reiner, 2014), which reduced the incidence of coronary events by only 30% (Fulcher et al., 2015). Thus more perspectives believe that additional treatment is needed to provide more effective coronary artery care, and drugs with endothelial protection may effectively inhibit the development of AS. Ginsenoside Rg3 is one of the important ginsenosides in ginseng, the pharmacological effects of which mainly focus on treatment of tumor related diseases. A study showed that Rg3 alleviated the adverse reactions of transcatheter arterial chemoembolization in patients with hepatocellular carcinoma, and prolonged the survival of patients (Zhou et al., 2016). Rg3 can restore endothelial dysfunction against cardiotoxicity by inhibiting oxidative damage and apoptosis *via* Akt-mediated activation of the NF-E2-related factor 2-antioxidant response element (Nrf2-ARE) pathway injured by adriamycin (Wang et al., 2015). Therefore, the effect of Rg3 on endothelial dysfunction and AS caused by dyslipidemia and whether related to regulated FAK-mediated pathways need for further investigation which provides important accordance for Rg3 applicate in anti-atherosclerosis.

Peroxisome proliferator-activated receptor gamma (PPARγ) is the target of clinical insulin sensitizers, which can protect diabetes and related complication, such as endothelial cell injury and vascular endothelial insulin resistance (IR) induced by high glycemic (Zhang et al., 2015). Research on the variance in gene expression profiles in the renal cortex of diabetic nephropathy rats treated with Rg3 showed that the PPAR signaling pathway was predominantly altered, indicating that PPAR is involved in mechanisms underlying Rg3 improving diabetic nephropathy (Wang et al., 2016). Evidence showed that PPARγ may also involve in lipid metabolism (Han et al., 2017), the obesity associated with chronic hypoxia can lead to severe endothelium-dependent diastolic dysfunction, resulting in obstructive sleep apnea (OSA), whereas the PPARγ agonist can significantly improve endothelial diastolic function and thus inhibit OSA (Zhang et al., 2017). Thus, whether Rg3 have effects on regulating PPARγ in dyslipidemia induced AS which interfere FAK-related pathways or not have yet to be explored. In this study, we take the ox-LDL-induced endothelial dysfunction and high fat diets (HFD) induced ApoE^−/−^ mice as the research objects to investigate the effect and possible mechanism of Rg3 on AS, which provide evidence for potential clinical application of Rg3 in the treatment of AS or concurrent cardiovascular diseases.

## Materials and Methods

### Reagents

HUVECs, THP-1 monocytes, and cell culture medium were purchased from Wuhan Procell Life Science and Technology (Wuhan, China). Rg3 (purity: > 98%, lot number: 20151201) was purchased from Yatai Pharmaceuticals (Changchun, China). Ox-LDL was purchased from Guangzhou Yiyuan Biotechnologies (Guangzhou, China). The BCA protein assay kit, radio immunoprecipitation assay (RIPA) lysis buffer, BCECF AM, QuickBlock™ Blocking Buffer for Western blot, and BeyoECL Plus were supplied by Shanghai Beyotime Biotechnology (Shanghai, China). GW9662 was purchased from MedChemExpress (Shanghai, China). Oil red O staining solution was from Sigma-Aldrich (Darmstadt, Germany). Horseradish peroxidase (HRP) Detection System and diaminobenzidine (DAB) were from ZSGB-BIO (Beijing, China). Low-density lipoprotein (LDL) assay kit (A113-1), high-density lipoprotein (HDL) assay kit (A112-1), total cholesterol (TC) assay kit (A111-1) and triglyceride (TG) assay kit (A110-1) were from Nanjing Jiancheng Bioengineering Institute (Nanjing, China). TRIzol^®^ was obtained from Thermo Fisher Scientific (Waltham, MA, USA). TransScript Green Two-Step qRT-PCR SuperMix was purchased from Beijing TransGen Biotech (Beijing, China). ApoE^−/−^ mice were purchased from the Beijing Vital River Laboratory Animal Technology (Beijing, China). High-fat diets were purchased from Research Diets Inc. (D12108, New Brunswick, NJ, USA). The anti-GAPDH monoclonal antibody (TA-08), HRP-conjugated anti-rabbit IgG (IH-0011) or anti-mouse IgG (IH-0031), as well as primers of monocyte chemotactic protein 1 (MCP-1), interleukin 6 (IL-6), interleukin 1β (IL-1β) and tumor necrosis factor α (TNF-α) were from Beijing Dingguochangsheng Biotechnology (Beijing, China). ICAM-1 antibody (no. 10831-1) was from Proteintech (Wuhan, China). PPARγ antibody (no. 2435), phosphor-FAK antibody (no. 3281), FAK antibody (no. 3285), VCAM-1 antibody (no. 13662, no. 32653), MMP-2 antibody (no. 13732) and MMP-9 rabbit mAb (no. 13667) were from Cell Signaling Technology (Danvers, MA, USA). Another ICAM-1 antibody (ab171123) and p-NF-kB antibody (ab86299) were from Abcam (Cambridge, U.K.).

### Cell Culture

HUVECs were cultured with endothelial cell culture medium (Ham’s F-12K) supplemented with 10% fetal bovine serum (FBS), 0.05 mg/ml endothelial cell growth supplement (ECGS), 0.1 mg/ml heparin, and 1% penicillin/streptomycin at 37°C and 5% CO_2_. The THP-1 cells were cultured with Roswell Park Memorial Institute (RPMI)-1640 medium supplemented with 10% FBS, 0.05 mmol/L β-mercaptoethanol, and 1% penicillin/streptomycin at 37°C and 5% CO_2_.

### MTT Assay

HUVECs in the logarithmic growth phase were dispersed by trypsinization and seeded into 96-well plates at a density of 4 × 10^4^ cells/ml and 200 μl/well overnight. HUVECs were treated with 0, 7.5, 15, or 30 μM Rg3 for 48 h in order to estimate whether this agent induced HUVEC injury. Subsequently, 20 μl MTT (5 mg/ml in PBS) solution was added into each well and incubated for 4 h. A total of 150 μl dimethyl sulfoxide was added to each well, and the plates were placed on a shaker for 10 min. The absorbance at 570 nm was measured with a microplate reader (SpectraMax Plus384, Molecular Devices, Sunnyvale, CA, USA). The percentage of surviving cells was calculated as a fraction of the negative control.

### Wound Healing

HUVECs were seeded into 6-well plates at 2 × 10^5^ cells/ml and 2 ml/well until the cell fusion rate reached 100%. Then, Rg3 was added for 24 h, and GW9662 (PPARγ-specific inhibitor) was added 2 h before Rg3. Subsequently, the cell layer was scratched with micro pipette tips perpendicular to the cell plane, and cells were washed with sterile PBS to ensure that the scratch clearance was visible. Subsequently, ox-LDL was added for another 24 h. All the reagents were prepared with medium containing 1% FBS, and fresh medium supplemented with 1% FBS was added in control as a blank. Images were photographed with a microscope (Olympus Corporation, Tokyo, Japan) before and after scratches in fixed positions, and the area between cells was calculated using Image J software (National Institutes of Health, Bethesda, MD, USA).

### Monocyte Adhesion Assay

HUVECs were seeded into 24-well plates at 2 × 10^4^ cells/ml and 500 μl/well, then treated as previous described. THP-1 cells were labeled with 5 μM BCECF AM, which were diluted with 1640 medium for 30 min at 37°C, avoiding light, supernatant of HUVECs culture medium were removed and the tagged THP-1 cells were added into 24 well plates at 5 × 10^5^ cells/ml for another 1 h at 37°C. Un-adhered cells were washed with PBS, pictures were captured with a fluorescence microscope, and the number of adhering THP-1 cells was calculated.

### Transwell Migration Assay

HUVECs were treated as previous described and harvested in 6-well plates. Then, HUVECs were resuspended to 1 × 10^5^ cells/ml with medium containing 1% FBS, 200 μl cell suspension were placed in the upper chamber of an insert (pore size = 8 μm). The lower chamber was filled with medium containing 10% FBS (600 μl) in 24-well plates. After 24 h of incubation, removed cells on the upper chamber of the filter with a cotton swab, the underside cells were fixed with 4% paraformaldehyde for 30 min at room temperature, and then stained with 0.1% crystal violet diluted with 20% ethanol for 15 min. Migrating cells were photographed under a light microscope and counted using Image J software in three independent fields for each group.

### Immunofluorescence

HUVECs were seeded into 24-well plates and treated as previous described. Then HUVECs were fixed with 4% polyformaldehyde and permeated with 0.3% Triton X-100. Subsequently, the HUVECs were blocked with 5% non-immune goat serum and incubated with p-NF-kB (1:250) primary antibody overnight at 4°C. HUVECs were then washed with PBS 3 times, incubated with fluorescein isothiocyanate (FITC)-labeled goat anti-rabbit IgG fluorescent antibody (1:1000) for 1 h, avoiding light. Finally, HUVECs were washed with PBS 3 times, the nuclei were stained with DAPI for 5 min, showing blue fluorescence, and p-NF-kB positive staining presented as green fluorescence, images were photographed under a fluorescence microscope.

### qRT-PCR Assay

Processed HUVECs in 6-well plates were harvested and lysed in TRIzol reagent, mixed with chloroform by gently swirling, and centrifuged to obtain the upper aqueous phase. Isopropyl alcohol was added, the cells were centrifuged for sedimentary RNA, then washed with 75% ethanol, centrifuged again, and then resuspended in diethyl pyrocarbonate (DEPC) water. The OD value ratio was detected to be 260 nm/280 nm and the ratio was 1.4:2.0. Subsequently, RNA was reverse-transcribed with oligo (dT) primers, and qPCR was conducted with gene-specific primers in the presence of SYBR Premix Ex Taq. qPCR was conducted for three independent experiments, using GAPDH as the housekeeping control. qPCR amplification was performed with 40 to 50 cycles (95°C, 5 s; 55°C, 15 s; 72°C, 10 s), and the oligonucleotide primer sets are as fllows: MCP-1 forward primer 5′-CTTCATTCCCCAAGGGCTCG-3′ and reverse primer 5′-GCTTCTTTGGGACACTTGCTG-3′; IL-6 forward primer 5′-TCAATGAGGAGACTTGCCTG-3′ and reverse primer 5′-GATGAGTTGTCATGTCCTGC-3′; IL-1β forward primer 5′-CTTCGAGGCACAAGGCACAA-3′ and reverse primer 5′-TTCACTGGCGAGCTCAGGTA-3′; TNF-α forward primer 5′-AGAACTCACTGGGGCCTACA-3′ and reverse primer 5′-GCTCCGTGTCTCAAGGAAGT-3′; GAPDH forward primer 5′-AGAAGGCTGGGGCTCATTTG-3′ and reverse primer 5′-AGGGGCCATCCACAGTCTTC-3′. The relative expression levels of the target gene were calculated using the 2^−ΔΔCt^ method.

### Western Blot Analysis

HUVECs were seeded into 6-well plates at 2 × 10^5^ cells/ml, which were treated with indicated concentrations of Rg3 and GW9662, respectively, and incubated with or without ox-LDL. Then HUVECs were harvested in RIPA lysis buffer containing moderate protease inhibitor on ice, and the protein concentration was determined with a protein assay kit. The cell extract was adjusted to equal protein concentrations, mixed with 6× loading buffer in a ratio of 1:5, boiled for 5 min, and then loaded onto polyacrylamide- sodium lauryl sulfate (SDS) gels. Subsequently, gel electrophoresis was conducted at 100 V, the proteins were transferred from gel to polyvinylidene fluoride membrane in an ice bath, the PVDF membrane was blocked with blocking buffer for 15 min. The membranes were then incubated with primary antibodies, including PPARγ antibody (1:1000), phosphor-FAK antibody (1:1000), FAK antibody (1:1000), ICAM-1 antibody (1:1000), VCAM-1 antibody (1:1000), MMP-2 antibody (1:1000), MMP-9 antibody (1:1000), and mouse anti-GAPDH monoclonal antibody (1:5000) at 4°C overnight. Finally, the bindings of target proteins were detected with secondary antibody conjugated to HRP (1:5000 dilution) and visualized using ECL chemiluminescence.

The whole aorta was removed completely and peeled off the adipose tissue around blood vessel. The aorta was cut into small pieces and harvested in RIPA lysis buffer containing moderate protease inhibitor and ultrasound homogenate, and the protein concentration was determined with a BCA protein assay kit. The tissue protein was extracted for detection by western blot analysis as mentioned methods, primary antibodies included PPARγ antibody (1:1000), phosphor-FAK antibody (1:1000), FAK antibody (1:1000). The second antibody incubation and visualization methods were the same as before. Finally, densitometric analysis of all the bands was conducted using Image J software.

### Atherosclerosis Animal Model Protocol

ApoE^−/−^ mice (eight-week-old males) were fed atherogenic chow (i.e., a high-fat diet with 40 kcal% fat, 1.25% cholesterol), and C57BL/6N mice (WT) were fed normal chow as the baseline control. After being fed atherogenic chow for 8 weeks, the ApoE^−/−^ mice were randomly divided into three groups (n = 8 each): model group (Mod), Rg3 15 mg/kg group, and Rg3 30 mg/kg group, and the mice were killed after 4 weeks with oral administration.

### Detection of Serum Lipid Proteins Levels, Histopathology, and Immunohistochemistry

At the end of the experiment, the rats were sacrificed after Rg3 treatment. Get the blood plasma for detecting LDL, HDL, TC, and TG levels in serum, all samples were measured according to the instruction utilizing a Hitachi 7150 Biochemical Autoanalyzer (Hitachi, Tokyo, Japan).

The mice were sacrificed and fixed on an anatomical plate. Then 75% ethanol was used to disinfect the mice abdomen, and the abdominal cavity was opened to exposed heart. The heart was gently clamped, and an infusion needle was inserted into the apex cordis. The right auricle was cut and perfused with 10 to 20 ml of 4% tissue cell stationary fluid. The other organs, except for the heart, aorta, and kidney, were removed, and the whole aorta was separated carefully along the spine to the iliac artery. Finally, the kidney was removed, and myocardial tissue and vascular adipose tissue were carefully exfoliated outside under a stereomicroscope, dissected longitudinally with precision scissors, and then fixed with 4% polyformaldehyde for 24 h. Subsequently, the aortas were washed with PBS for 10 min, then washed with 60% isopropanol for 5 s at room temperature, and stained in oil red O for 2 h, avoiding light. After staining, the aortas were transferred to 60% isopropanol for decolorization and washed for 5 min, then washed with PBS for 5 min to remove isopropanol. Oil red O stained the lipid-rich plaque to red and lightened the color of the non-plaque area. The aortas were smoothed on a black background and photographed. The total area and the red plaque area were calculated using Image J software.

The aortic arch was dissected, removed, fixed in 4% polyformaldehyde, embedded in paraffin, and prepared into 5-μm-thick sections. The sections were stained with hematoxylin and eosin (H&E) for plaque morphology. After blocking in 5% bovine serum albumin (BSA) in PBS, the sections were incubated with primary antibodies overnight at 4°C, and then incubated with an HRP Detection System. Detection was subsequently performed using (DAB). Staining results were all observed under a microscope and photographed and analyzed utilizing Image-Pro Plus 6 analysis software (Media Cybernetics, Silver Spring, MD, USA).

### Statistical Analysis

Statistical analysis was performed using the SPSS 22.0 statistical package (SPSS, Inc., Chicago, IL, USA). The results are expressed as the mean ± standard deviation. Statistical differences among all groups were evaluated using one-way analysis of variance (ANOVA) followed by the Tukey Honest Significant Difference test. *P* < 0.05 was considered to be a statistically significant difference.

## Results

### Rg3 Ameliorate HUVECs Function Stimulated With ox-LDL

**Figure 1:**
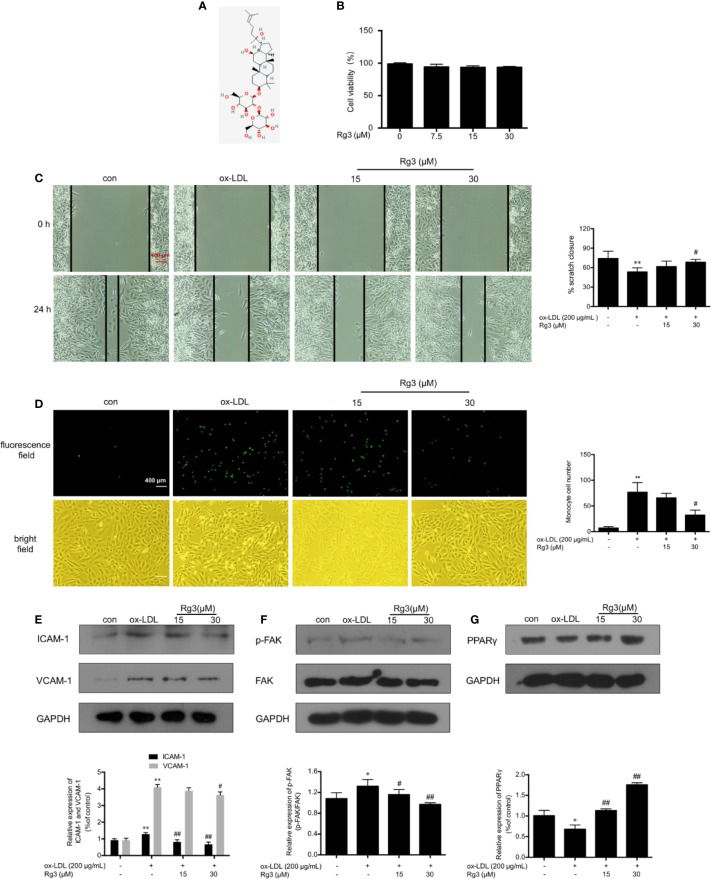
Rg3 ameliorated HUVECs function and down-regulated expression of adhesive molecules stimulated with ox-LDL. **(A)** The structure of 20(R)-Rg3, molecular formula: C42H72O13, molecular weight: 785.025 g/mol (U.S. national library of medicine). **(B)** MTT assay of different Rg3 concentration on cell viability. **(C)** After treatment with Rg3, cultured HUVECs had a scratch placed in the middle of the six-well plates as a basal control (0 h); then, ox-LDL (200 μg/ml) was added for 24 h. Observed under a microscope and photographed at 0, 24 h in same position. % scratch closure = (the area at 0 h − the area at 24 h)/the area at 0 h. **(D)** Representative images of labeled THP-1 cells adherent to HUVECs. 2′,7′-bis-(2-carboxyethyl)-5-(and-6)-carboxyfluorescein, acetoxymethyl ester (BCECF AM)-labeled THP-1 showed green fluorescence. The statistical number of THP-1 cells adherent to HUVECs. **(E–G)** The expression of ICAM-1, VCAM-1, p-FAK, and PPARγ were determined by western blotting, the phosphorylation of FAK is expressed as the ratio of phosphorylated-FAK to total FAK, GAPDH was used as a loading control. Values are expressed as the mean ± s.d. from three independent experiments; magnification, ×100; scale bar = 400 μm. ^*^P < 0.01 vs. control group; ^**^P < 0.01 vs. control group; ^#^*P* < 0.05 vs. ox-LDL group; ^##^*P* < 0.01 vs. ox-LDL group.

The structure of Rg3 is shown in **Figure 1A**. We examined effect of Rg3 on cell viability by MTT assay to ensure the Rg3 concentration were appropriate with no damage to HUVECs, then we considered the 15, 30 μM Rg3 for further experiments **Figure 1B**). To determine whether Rg3 protects endothelial function stimulated by ox-LDL, we first investigated the effects of Rg3 on endothelial cell healing, as shown in **Figure 1C**, ox-LDL inhibited endothelial cell healing after scratching, whereas 30 μM Rg3 significantly reversed HUVECs healing repressed by ox-LDL. Meanwhile, there were much increased THP-1 adhesion to HUVECs after ox-LDL stimulated than control **Figure 1D**), whereas 30 μM Rg3 significantly reversed THP-1 adhesion, indicating that Rg3 has a protective effect on endothelial function disrupted by ox-LDL.

### Rg3 Down-Regulated Expression of Adhesive Molecules in HUVECs Stimulated by Ox-LDL

ICAM-1 and VCAM-1 are the main regulatory factors recruiting monocytes, ox-LDL can remarkably enhanced the expression of ICAM-1 and VCAM-1 in HUVECs **Figure 1E**), Rg3 decreased the expression of adhesion factors to varying degrees, and the effect of 30 μM Rg3 was much better. Studies showed that FAK activation contribute to the expression of adhesion molecules, relating to the formation of atherosclerotic plaques (Yurdagul et al., 2016). As seen in **Figure 1F**, ox-LDL severely enhanced FAK phosphorylation, whereas 15 and 30 μM Rg3 significantly inhibited the effect of ox-LDL on FAK activation.

### Rg3 Protected HUVECs From Ox-LDL Activated FAK Signaling Pathway *via* Up-Regulating PPARγ

**Figure 2:**
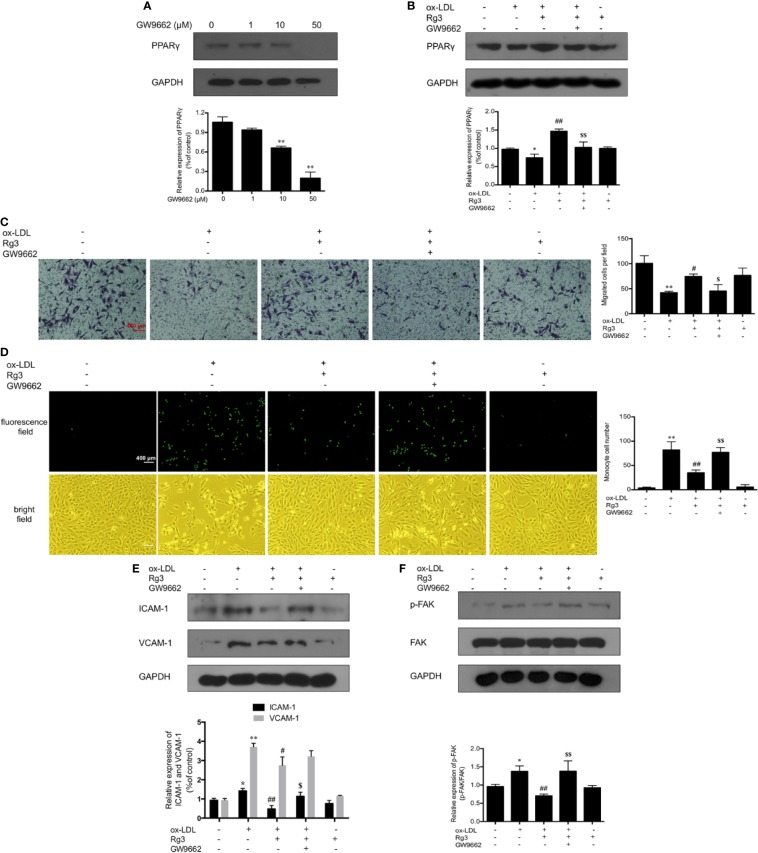
Rg3 protect HUVECs from ox-LDL activated FAK signaling pathway *via* up-regulating PPARγ. **(A)** The effect of different concentrations of GW9662 (0, 1, 10, and 50 µM) on PPARγ expression. **(B)** GW9662 (10 µM) restrained the effect of Rg3 (30 μM) on PPARγ expression in ox-LDL-simulated HUVECs. **(C)** GW9662 (10 µM) reversed the effect of Rg3 (30 μM) on HUVECs migration, the statistical number of migrated HUVECs were calculated. **(D)** GW9662 (10 µM) reversed the effect of Rg3 (30 μM) on THP-1 adhesion to HUVECs, the THP-1 cells adherent to HUVECs were calculated. **(E, F)** The expression of ICAM-1, VCAM-1, p-FAK, and PPARγ were determined by western blotting. Magnification, ×100; scale bar = 400 μm. Values are expressed as the mean ± s.d. from three independent experiments. ^*^P < 0.05 vs. control group; ^**^P < 0.01 vs. control group; ^#^*P* < 0.05 vs. ox-LDL group; ^##^*P* < 0.01 vs. ox-LDL group; ^$^*P* < 0.05 vs. Rg3 + ox-LDL group; ^$$^*P* < 0.01 vs. Rg3 + ox-LDL group.

As shown in **Figure 1G**, the expression of PPARγ in ox-LDL-stimulated HUVECs was significantly inhibited, whereas pretreatment with Rg3 significantly reversed the ox-LDL effect on PPARγ. Here, we applied GW9662 to evaluate whether Rg3 regulates the FAK-mediated adhesion dependent pathway in ox-LDL stimulated HUVECs *via* up-regulating PPARγ. As shown in **Figure 2A**, in HUVECs pretreated with 10 µM GW9662, the expression of PPARγ was significantly inhibited, GW9662 significantly inhibited the up-regulation of PPARγ by Rg3 (**Figure 2B**). Migration assay can also evaluate the cell healing ability on the other hand, the results showed that ox-LDL repressed HUVECs migration ability obviously, and Rg3 significantly reversed the ox-LDL effect, while GW9662 inhibited the effect of Rg3 on HUVECs migration (**Figure 2C**), as well as inhibited the effect of Rg3 on anti-monocyte adhesion (**Figure 2D**). GW9662 repressed the effect of Rg3 on the down-regulating expression of ICAM-1 and VCAM-1, which showed more significance on ICAM-1 (**Figure 2E**) and suppressed down-activation of FAK by Rg3 (**Figure 2F**), notably, adhesive molecules expression in HUVECs, which were treated with 30 μM Rg3 alone, did not differ from normal cells. The above results show that the protective effect of Rg3 on FAK-mediated adhesion dependent pathway may related to up-regulating PPARγ expression in ox-LDL stimulated HUVECs.

### Rg3 Repressed FAK Mediated Atherogenic Molecules *via* Up-Regulating PPARγ in Ox-LDL Stimulated HUVECs

**Figure 3:**
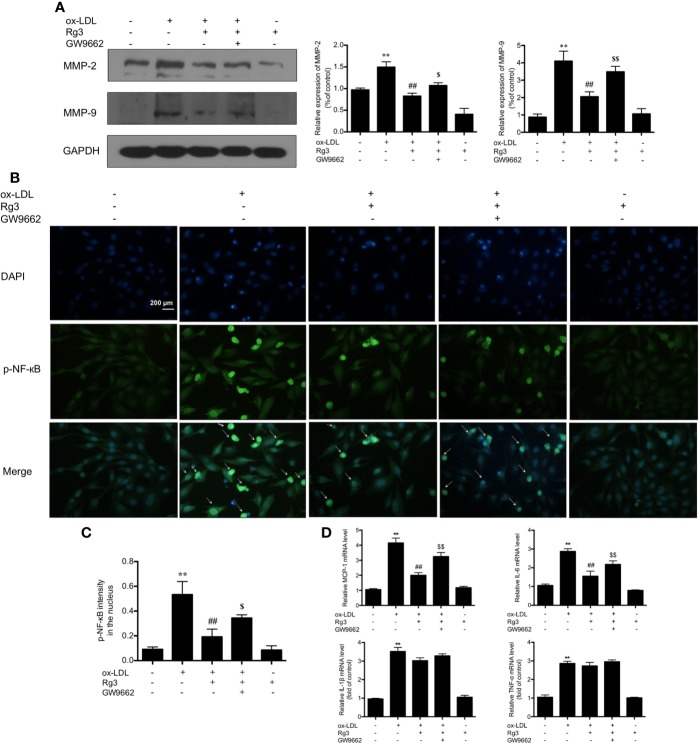
Rg3 repressed FAK mediated atherogenic molecules *via* up-regulating PPARγ in ox-LDL stimulated HUVECs. **(A)** The expression of MMP-2 and MMP-9 were determined by western blotting. **(B)** The phosphorylated-nuclear factor-kappa B (phosphorylated-NF-κB) activation in HUVEC nucleus; p-NF-κB showed green fluorescence and nuclear stained blue fluorescence by 4′,6-diamidino-2-phenylindole (DAPI). Magnification, ×200; scale bar = 200 μm. **(C)** Quantification above background levels of phosphorylated-NF-κB intensity in the nucleus. The data were presented as mean ± s.d. **(D)** The mRNA levels of MCP-1, IL-6, IL-1β, and TNF-α in each group. GAPDH was used as a loading control, the fold values compared to control are expressed as the mean ± s.d. from three independent experiments. ^**^P < 0.01 vs. control group; ^##^*P* < 0.01 vs. ox-LDL group; ^$^*P* < 0.05 vs. Rg3 + ox-LDL group; ^$$^*P* < 0.01 vs. Rg3 + ox-LDL group.

Activation of FAK can sensitize the non-adhesive-dependent atherogenic matrix metalloproteinases pathway (MMP) (Alford et al., 2017), including MMP-2 and MMP-9, which can stimulate the vulnerability of atherosclerotic plaques (Seifert et al., 2018), our study showed the same trend of MMP-2/9 in ox-LDL stimulated HUVECs, and Rg3 inhibit the MMP-2/9 expression, while GW9662 inhibited the effect of Rg3 on MMP-2 and MMP-9 (**Figure 3A**). Furthermore, the activation of NF-κB into the nucleus increased significantly in HUVECs after stimulating with ox-LDL, and the nucleus sustained more damage than normal HUVECs (**Figures 3B, C**). Pretreated with 30 μM Rg3, the activation of NF-κB and nucleus damage were significantly suppressed. GW9662 repressed the effect of Rg3. HUVECs treated with 30 μM Rg3 alone did not differ from normal cells. The mRNA levels of inflammatory factors, including MCP-1, IL-6, IL-1β, and TNF-α in ox-LDL-stimulated HUVECs significantly increased; when pretreated with 30 μM Rg3, levels of MCP-1 and IL-6 mRNA significantly decreased, whereas GW9662 repressed the effect of Rg3, but IL-1β and TNF-α mRNA were not significantly improved by Rg3 (**Figure 3D**), and stimulated Rg3 alone was nearly similar to normal HUVECs.

### Rg3 Restrained the Formation of Atherosclerosis in ApoE^−/−^ Mice

**Figure 4:**
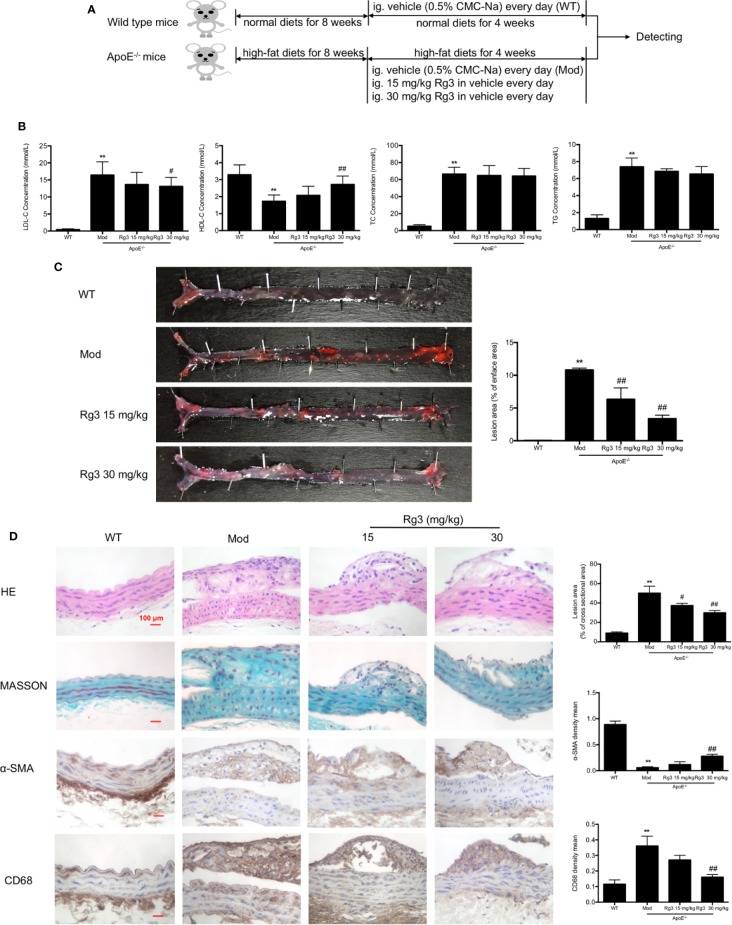
Rg3 restrained the atherosclerosis in ApoE^−/−^ mice. **(A)** The time schedule of this *in vivo* study. **(B)** The levels of LDL, HDL, TC, TG in serum of each group mice (n=8). **(C)** Oil Red O Staining on total aorta. Lesion area (%) = (red staining area/total area) of aorta × 100%. **(D)** Representative HE, MASSON, and immunohistochemistry staining of aortic root cross sections. Values are expressed as the mean ± s.d. Magnification, ×400; scale bar = 100 μm. ^**^P < 0.01 vs. control; ^#^*P* < 0.05 vs. model group; ^##^*P* < 0.01 vs. model group.

The AS model preparation and administration process are shown in **Figure 4A**. Lipoproteins in serum of model mice showed significant abnormalities, the levels of LDL, TC, TG were striking higher than normal mice, and the level of HDL was much lower (**Figure 4B**), while Rg3 can decrease LDL level and increase HDL level in ApoE^−/−^ mice, especially the effect of high dose Rg3 was more remarkable. Oil Red O staining results showed that high-fat diet led to a large amount of atherosclerotic plaque deposition in the inner surface of blood vessels than in mice fed a normal diet, indicating the successful replication of atherosclerosis model. Plaque in the aortic root showed an especially large area, and in the thoracic aorta and below, there was a punctate scattered distribution, whereas the plaque areas in the mice orally treated with Rg3 were much lighter and were concentration-dependent compared with those in the model group (**Figure 4C**). The cross-section lesion areas in aortic root of model mice were more serious than WT mice, and remarkably decreased in the Rg3-treated group (**Figure 4D**). Meanwhile, in aorta, the relative content of collagen fibers and α-smooth muscle actin (α-SMA) were strikingly lower in the model group than the WT group, whereas they were higher in the Rg3-treated group, especially in high-dose Rg3 group, as well as the CD68 content was strikingly higher in model than WT and higher in Rg3-treated group (**Figure 4D**). The results showed that Rg3 can significantly inhibit the formation of atherosclerotic plaques and improve lipoprotein abnormality in ApoE^−/−^ mice with HFD.

### Rg3 Repressed Atherosclerosis Related to Inhibit Adhesion Molecules in intima *via* Regulating PPARγ/FAK Signaling Pathway in ApoE^−/−^ Mice

**Figure 5:**
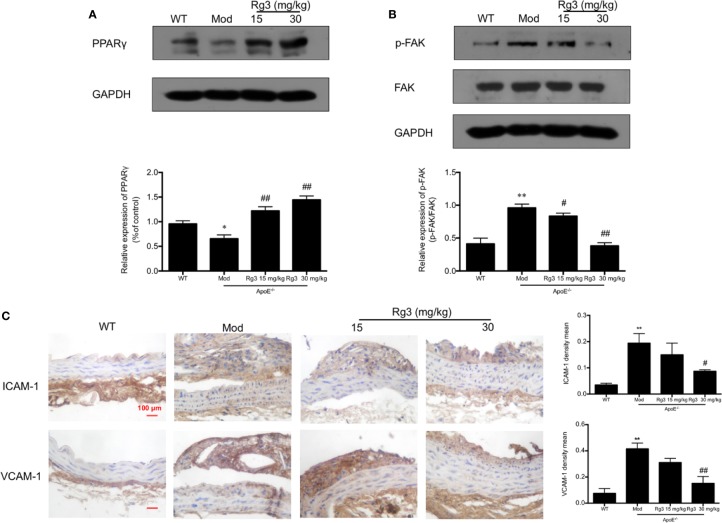
Rg3 repressed atherosclerosis and adhesion molecules in intima *via* regulating PPARγ/FAK signaling pathway in ApoE^−/−^ mice. **(A, B)** The expression of p-FAK and PPARγ in aorta by Western blotting. **(C)** Representative images of ICAM-1 and VCAM-1 immunohistochemical staining of aortic root cross sections. Values are expressed as the mean ± s.d. from three independent experiments. Magnification, ×400; scale bar = 100 μm. ^*^P < 0.05 vs. control; ^**^P < 0.01 vs. control; ^#^*P* < 0.05 vs. model group; ^##^*P* < 0.01 vs. model group.

Our results showed that the phosphorylation of FAK were significantly higher in aorta of the model group than the WT group, while the expression of PPARγ were lower in the model group. Conversely, and the phosphorylation of FAK were lower and PPARγ were higher in the Rg3-treated group (**Figures 5A, B**). Furthermore, the expression of ICAM-1 and VCAM-1 were significantly higher in aortic intima of the model group than the WT group, and lower in Rg3-treated group, especially significantly lower in Rg3 high dose group (**Figure 5C**). These results indicate that Rg3 may inhibit adhesion molecules in intima by regulating PPARγ/FAK in ApoE^−/−^ mice.

## Discussion

Atherosclerosis is a chronic inflammatory disease and different cells are involved in the disease processes, including endothelial cells (Paone et al., 2019), monocytes (Escarcega et al., 2018), macrophages (Biessen and Wouters, 2017), and smooth muscle cells (Lao et al., 2015). The latest epidemiological statistics in 2019 show that dyslipidemia remains a major risk factor for atherosclerotic cardiovascular disease (Egom et al., 2019). Dyslipidemia cover a wide range of lipoprotein abnormalities, including elevated levels of low-density lipoprotein cholesterol, total cholesterol, and total triglyceride, as well as decreased levels of high density lipoprotein cholesterol, especially the LDL receptors were firmly up-regulated in cholesterol metabolism (Goldstein and Brown, 2015), thus LDL can be ingested and modificated into ox-LDL exhibit a variety of proatherogenic properties on vascular cultured cells (Salvayre et al., 2016), and the intervention of ox-LDL for vascular cells in study of AS *in vitro* has been widely used. At the early stage of AS, ox-LDL preferentially accumulate at the arterial curvature and lead to endothelial dysfunction, including recruiting monocyte adhesion to the vascular endothelium by promoting endothelial cell ligands expression, such as VCAM-1 and ICAM-1 (Mestas and Ley, 2008), meanwhile, ox-LDL damage endothelial ability of healing which can prevent inflammation cells infiltrating into the endothelium to avoid damage to other vascular cells, thus many studies consider that the ox-LDL damage to endothelium is initial step of AS, and has important significance in study of anti-atherosclerosis. We used ox-LDL stimulated HUVECs to simulate the occurrence of early atherosclerosis and the healing ability of HUVECs was weakened, notably, the quantity of monocyte adhesion to endothelial cells significantly increased, and Rg3 significantly enhanced healing ability and inhibited monocyte adhesion to HUVECs stimulated with ox-LDL. According to Fan’s study (Fan et al., 2016), they reported that the C_max_ of Rg3 reached approximately 100 μmol/L in normal rats plasma after a single oral administration of Rg3, while that of tumor bearing rats was close to 46 μmol/L, indicating that it is possible for physiological concentration of Rg3 after oral administration to reach the higher concentration of Rg3 (30 μmol/L) applied in our study *in vitro*; meanwhile, in Jin’s recently pharmacokinetic studies (Jin et al., 2019), Rg3 was detected as one of the 13 major ginsenosides in human plasma after repeated oral administration of red ginseng extract for two weeks, the daily intake amount of Rg3 from red ginseng extract was 7.9 mg/day, and the C_max_ of Rg3 in human plasma reached 8.7 ng/ml (approximately equal to 13 μmol/L) which is close to our lower concentration of Rg3 (15 μmol/L), suggesting that the concentration of Rg3 we used *in vitro* is significant for the study of Rg3 pharmacology. Investigations have shown that Rg3 can reverse the M1 polarization to the M2 phenotype in diabetic conditions to repress the occurrence of diabetes-complicated atherosclerosis (Guo et al., 2018), our further experiments confirmed that HFD for 12 weeks lead to plenty of atherosclerotic plaque formation in aorta of ApoE^−/−^ mice, accompanied by abnormal lipoprotein profiles in serum, while Rg3 decreased LDL level and increased HDL level in serum of ApoE^−/−^ mice, inhibited atherosclerotic plaque formation obviously. Furthermore, the arterial wall contains a large amount of collagens, mainly including collagens I, III, V, and XIII (specific existence and endothelial cells) to ensure the elasticity of blood vessels, and maintain the stability of AS plaque, the decrease of collagens can make stable plaque become unstable plaque, then increase the risk of thrombosis (Copes et al., 2019), our results showed aortic collagens contents of AS mice were decreased, while significantly increased in Rg3 treated mice; it is also noted the expression of collagen in atherosclerotic plaques of AS mice increased significantly, study revealed that collagen II contents which mainly exist in cartilage would be increased in atherosclerotic plaques where cholesterol clefts and crystals were present, and relate to lipid core calcification (Kuzan et al., 2017). Meanwhile, Rg3 significantly decreased macrophage aggregation, increased contractile vascular smooth muscle cells (the marker protein is α-SMA) in mice aorta, these results suggest that Rg3 potentially protects against AS caused by abnormal lipid metabolism.

FAK was discovered as a substrate of the oncogene product of the sarcoma virus that exists in many cancer cells (Mousson et al., 2018), it is a key molecule that promotes the adhesion of cancer cells to adjacent organs by accelerating the expression of adhesion molecules, resulting in tumor metastasis (Leung et al., 2015). Increasing numbers of studies have shown that FAK may exist in non-cancer cells, participating in other pathological processes related to adhesion. FAK activation can promote the adhesion of macrophages to the extracellular matrix (ECM), which aggravates fibrosis-related diseases (Digiacomo et al., 2017). A study demonstrated FAK-mediated monocyte adhesion to endothelial cells by up-regulating VCAM-1 induced by ox-LDL (Yurdagul et al., 2016), which is consistent with our experimental results, the expression of VCAM-1 and ICAM-1, and activation of FAK were increased in HUVECs under ox-LDL stimulation *in vitro*, pretreated with Rg3 in ox-LDL stimulated HUVECs significantly suppressed FAK activation and VCAM-1 and ICAM-1 expression in a dose-dependent manner, indicating that Rg3 may play a role in protecting endothelial function by repressing FAK activation. Furthermore, FAK is highly conserved during evolution and widely expressed in different cells, FAK protein levels are increased in adipocytes of insulin-resistant mice which relate to adipose tissue expansion, leading to obese (Luk et al., 2017), FAK also can be activated by TNF-α in vascular smooth muscle cells which is involved in the process of atherosclerosis restenosis (Yu et al., 2018), suggesting that activation of FAK may increase not only in abnormal vascular endothelial cells and smooth muscle cells, but also in whole aorta of atherosclerotic ApoE^−/−^ mice (Chen et al., 2018). In this study, activation of FAK was remarkable increased in gross aorta of ApoE^−/−^ mice, while the ICAM-1 and VCAM-1 expression were increased mainly derived from vascular endothelium, and study confirmed that ICAM-1 and VCAM-1 were the endothelial-derived ligands may recruit monocytes, dendritic cells, T cells by binding to the integrin very late antigen-4 and lymphocyte function-associated antigen-1 expressed in these immune cells (Wu et al., 2017). Rg3 can significantly inhibit the activation of aortic FAK and the expression of ICAM-1 and VCAM-1 in vascular endothelium which may be a key mechanism of Rg3 against AS in HFD-induced ApoE^−/−^ mice; and Cho et al. showed that Rg3 significantly inhibited lipopolysaccharide (LPS)-induced VCAM-1 and ICAM-1 expression, and prevented the IκBα degradation in C57BL/6 mice to anti-inflammation in AS (Cho et al., 2014).

PPARγ seems to produce improvement in the epithelial-to-mesenchymal transition and tubulointerstitial fibrosis by up-regulating FAK activation to ameliorate diabetic nephropathy (Yan et al., 2019). PPARγ agonists can negatively regulate platelet activation and thrombus formation by reducing components of αIIbβ3 signaling, including FAK activation (Unsworth et al., 2016). According to the previous studies, PPARγ may be a key regulator of FAK activation in AS; our results indicated that the expression of PPARγ decreased in ox-LDL-treated HUVECs, as well as in aorta of HFD fed ApoE^−/−^ mice, and Rg3 significantly up-regulated PPARγ expression both in HUVECs and AS mice. Furthermore, the expression of PPARγ can be inhibited by ox-LDL in vascular smooth muscle cells which is disadvantageous to ensure the function and structure of blood vessels (Unsworth et al., 2016), while whether the mechanisms of Rg3 on FAK dependent processes in AS is related to regulating PPARγ, especially in endothelial cells are still not well established, thus we pretreated ox-LDL stimulated HUVECs with GW9662 (a specific inhibitor of PPARγ), and the previously shown endothelial protective effects of Rg3 were inhibited, suggesting that Rg3 may protect from AS by inhibiting the FAK-dependent adhesion pathway by up-regulating PPARγ expression in endothelial cells. FAK activation can also activate non-adhesion-dependent signals in HUVECs to promote the development of AS. MMP is one of the important pathways significantly affected by FAK activation (Alford et al., 2017). MMP-2/9 can increase the instability of atherosclerotic plaque (Seifert et al., 2018); our results showed that the expression of MMP-2 and MMP-9 were enhanced in ox-LDL-stimulated HUVECs, and GW9662 repressed the inhibitory effect of Rg3 on MMP-2 and MMP-9, study showed that NF-κB activation was achieved by up-regulating FAK expression *via* RSK/IKKβ activation in ox-LDL-stimulated endothelial cells (Yurdagul et al., 2016), and Rg3 significantly inhibited NF-κB activation by regulating the PPARγ/FAK pathway in ox-LDL-stimulated endothelial cells, along with repressed IL-6 and MCP-1 mRNA levels which is inflammatory factor promoting monocyte infiltration. Guo et al. confirmed that Rg3 can inhibit the inflammatory macrophages infiltration and injury of induced by persistent hyperglycemia by up-regulating the expression of PPARγ (Guo et al., 2018), and PPARγ is the target of anti-diabetes, our study further confirmed Rg3 not only improve hyperglycemia complicated with AS, but also has a good effect on AS caused by dyslipidemia by regulating PPARγ. Furthermore, Rg3 regulated PPARγ/FAK pathway accompanied with the repression of inflammatory factors-NF-κB in our study, and in addition to FAK-mediated pro-inflammatory NF-κB in AS (Chen et al., 2018), IκB kinases (IKKs) activation which triggered by assembly of multiprotein complexes starting a cascade of protein phosphorylation, non-degradative ubiquitination, and higher-order oligomerization directly mediates NF-κB activation (Fusella et al., 2019); meanwhile, miRNAs may regulate the expression of adhesion molecules through NF-κB pathway, which directly controls their transcription (Zhong et al., 2018), thus prompting that we should further focus on the research to make clear how Rg3 inhibit NF-κB and related pathways in endothelial cell, and further verify our results by using endothelial specific PPARγ deficient mice, so as to provide more basis for the application of Rg3 in AS.

In conclusion, our study initially revealed that Rg3 can ameliorate the healing and anti-monocyte adhesion ability of endothelial cells under high ox-LDL environment and alleviate atherosclerotic plaque induced by HFD in ApoE^−/−^ mice, which are related to regulating the ICAM-1, VCAM-1 expression in HUVECs and vascular endothelium *via* PPARγ/FAK pathway, indicating that Rg3 has good protective effect on atherosclerosis caused by dyslipidemia and is related to its protective effect on endothelium. Moreover, FAK-activated non-adhesion-dependent atherosclerotic pathways in endothelial cells can be repressed by Rg3. Our study provides stronger evidence for the anti-atherosclerosis effect of Rg3, which may have good potential value in preventing the formation of atherosclerotic plaques caused by dyslipidemia, or when lipid-lowering therapy is not effective in controlling the occurrence and development of AS, Rg3 has a good application prospect.

## Data Availability Statement

The datasets generated for this study can be found in the GeneBank containing MCP-1 primer (NM_002982.4), IL-6 primer (NM_001371096.1), IL-1β primer (NM_000576.3), TNF-α primer (NM_000594.4).

## Ethics Statement

All *in vivo* protocols involving animal care and experiments complied with the Guide for Care and Use of Laboratory Animals published by the United States National Institutes of Health (NIH Publication, 8th Edition, 2011) and the Animal Management Rules of the Chinese Ministry of Health (Document no. 55, 2001). All the *in vivo* experiments were approved by the Animal Experimental Ethical Inspection Committee of Jilin University School of Pharmaceutical Sciences (ethical permission code 20180024 and permission date September 30, 2018). All the *in vitro* experimental protocols were approved by the Laboratory of Cardiovascular and Tumor Pharmacology Research, School of Pharmaceutical Sciences, Jilin University, China.

## Author Contributions

JG, LF, and HX contributed conception and design of the study. JG, DS, and HX organized the database, performed the statistical analysis, and wrote the first draft of the manuscript. WF and ZL carried out the cell culture studies, molecular biology experiments, data collection, and statistical analysis. XY, YL, MS, PY, and XL participated in the animal experiments, data collection, and statistical analysis. HX and WF read and revised the entire manuscript. All authors contributed to manuscript revision, read, and approved the submitted version.

## Funding

This research was funded by National Natural Science Foundation of China (81473378), Natural Science Foundation of Jilin Province (20170101002JC) and Natural Science Foundation of Jilin Province (20190201262JC).

## Conflict of Interest

Author LF was employed by company Dalian Fusheng Pharmaceutical Co., Ltd and company Jilin Yatai Pharmaceutical Co., Ltd.

The remaining authors declare that the research was conducted in the absence of any commercial or financial relationships that could be construed as a potential conflict of interest.
