# Supplementary figures and images for: Ginsenoside Rg3 Alleviates ox-LDL Induced Endothelial Dysfunction and Prevents Atherosclerosis in ApoE−/− Mice by Regulating PPARγ/FAK Signaling Pathway
Source: Front Pharmacol. 2020 Apr 22;11:500. doi: 10.3389/fphar.2020.00500 (PMC7188907; doi:10.3389/fphar.2020.00500)

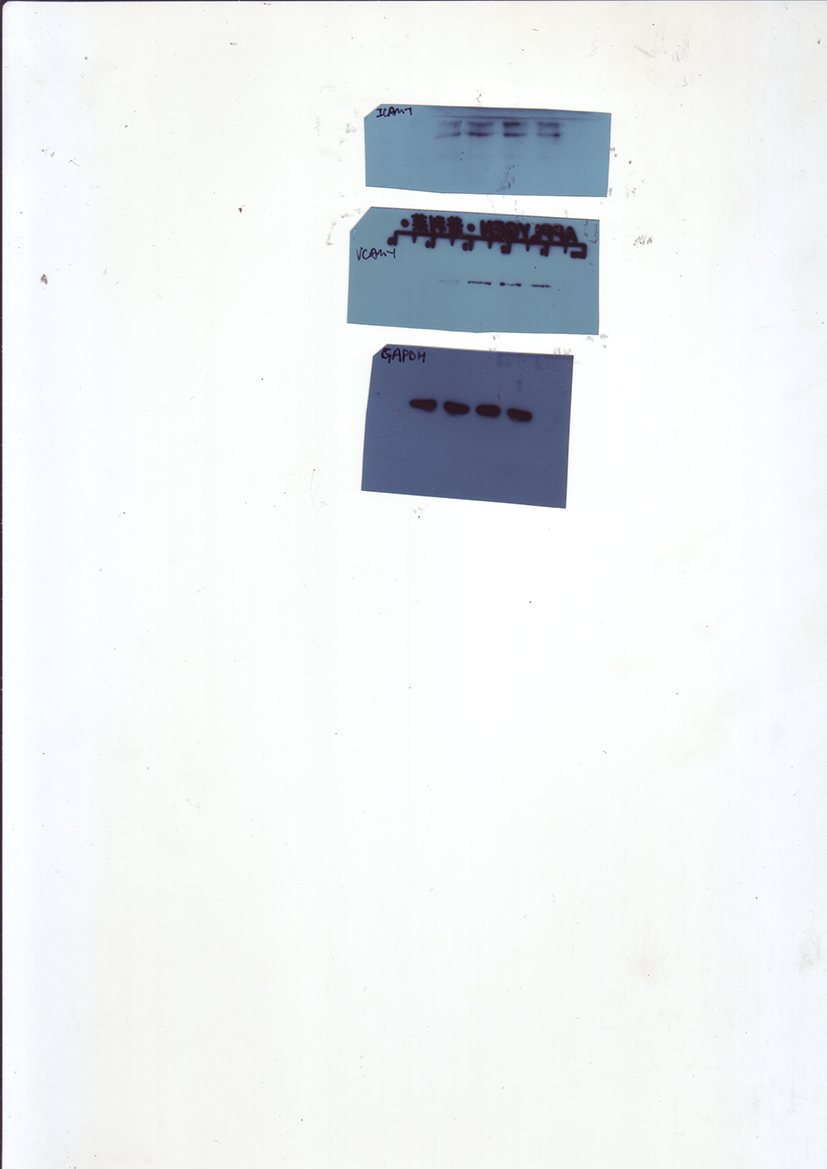

Supplement: Supplementary file 1 [file DataSheet_1.zip › Supplementary materials/Original images of Western blots(Fig 1F).tif]

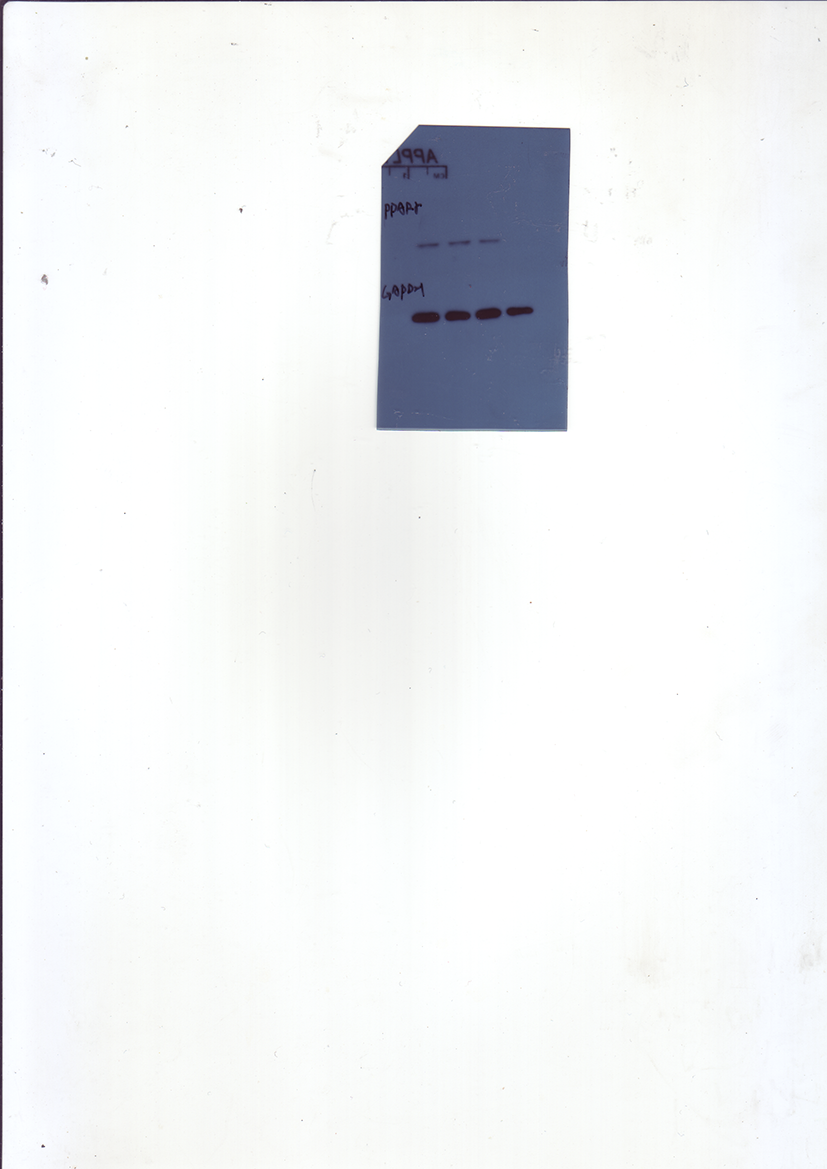

Supplement: Supplementary file 1 [file DataSheet_1.zip › Supplementary materials/Original images of Western blots(Fig 2A).tif]

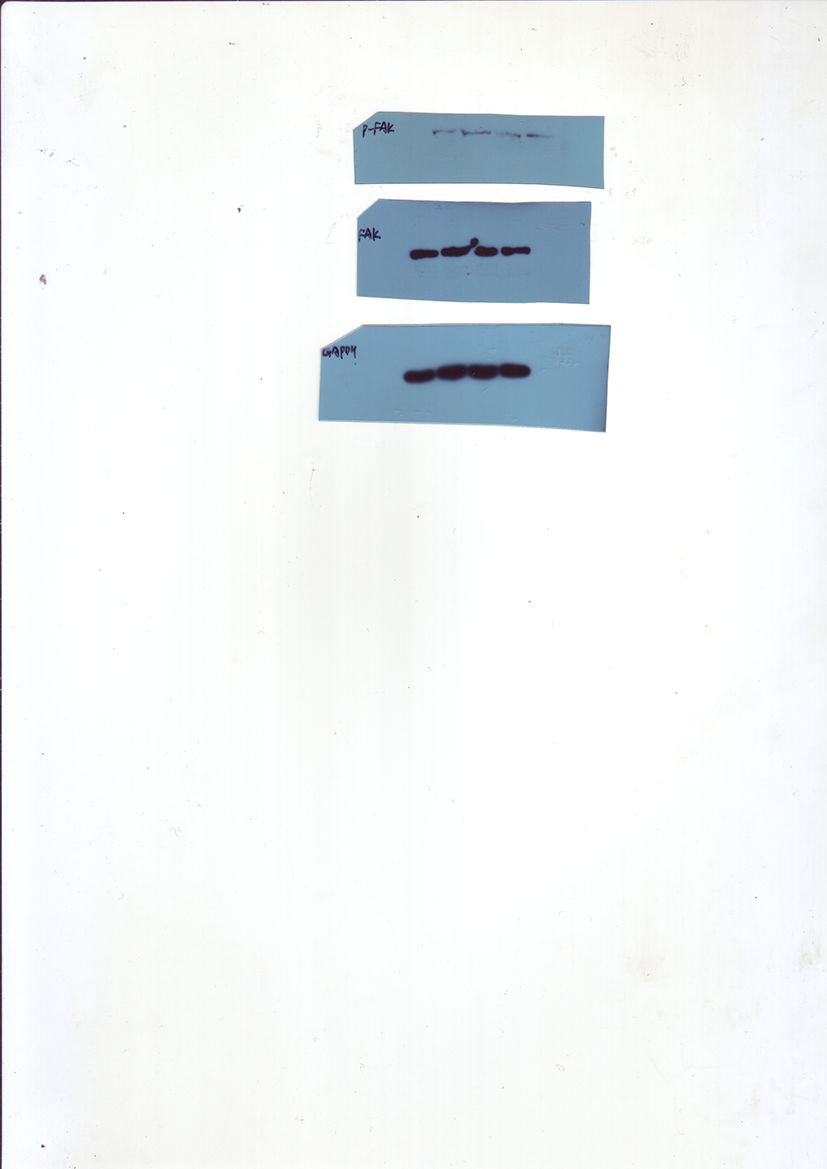

Supplement: Supplementary file 1 [file DataSheet_1.zip › Supplementary materials/Original images of Western blots(Fig 1G).tif]

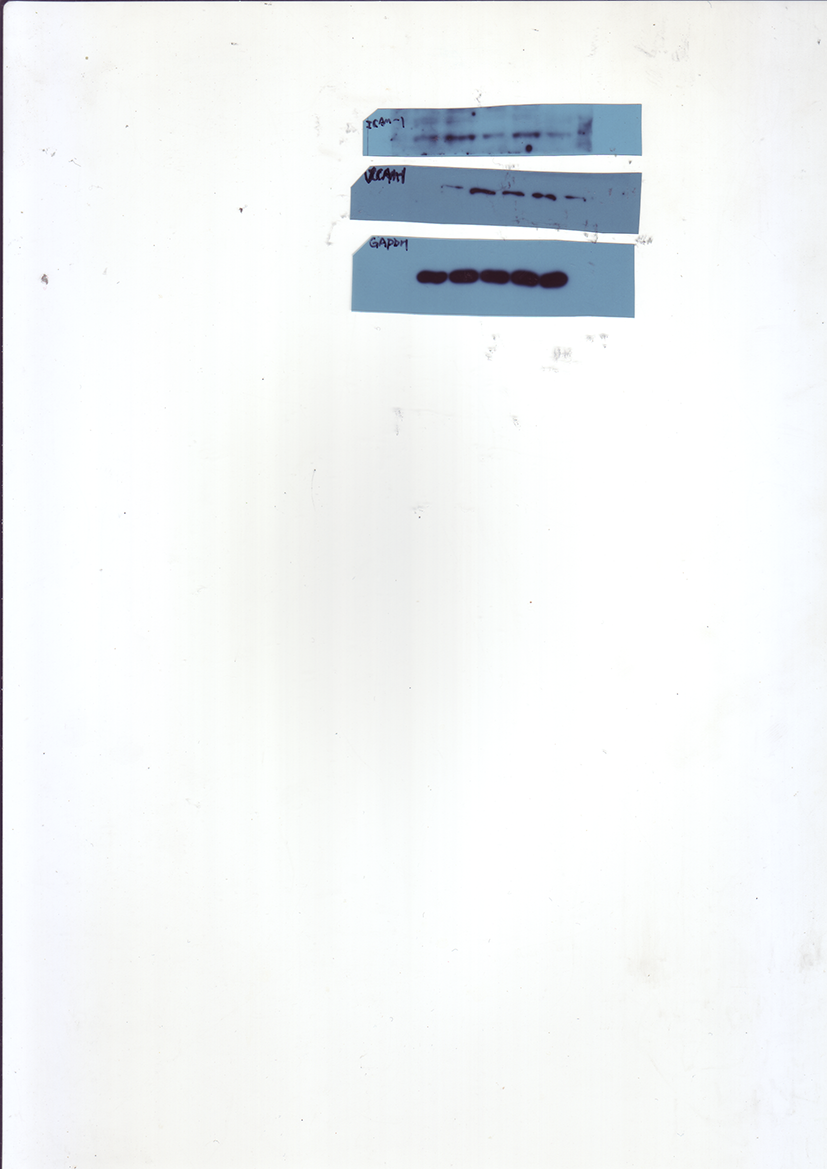

Supplement: Supplementary file 1 [file DataSheet_1.zip › Supplementary materials/Original images of Western blots(Fig 2F).tif]

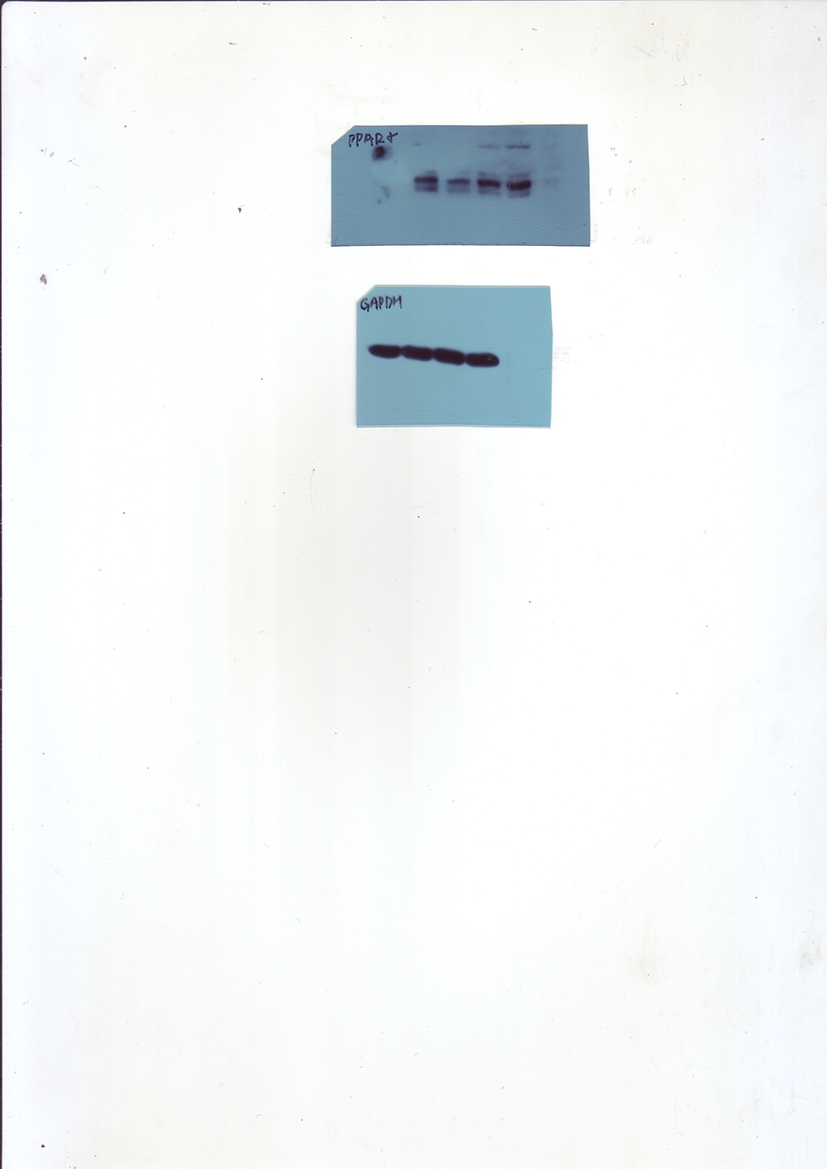

Supplement: Supplementary file 1 [file DataSheet_1.zip › Supplementary materials/Original images of Western blots(Fig 5A).tif]

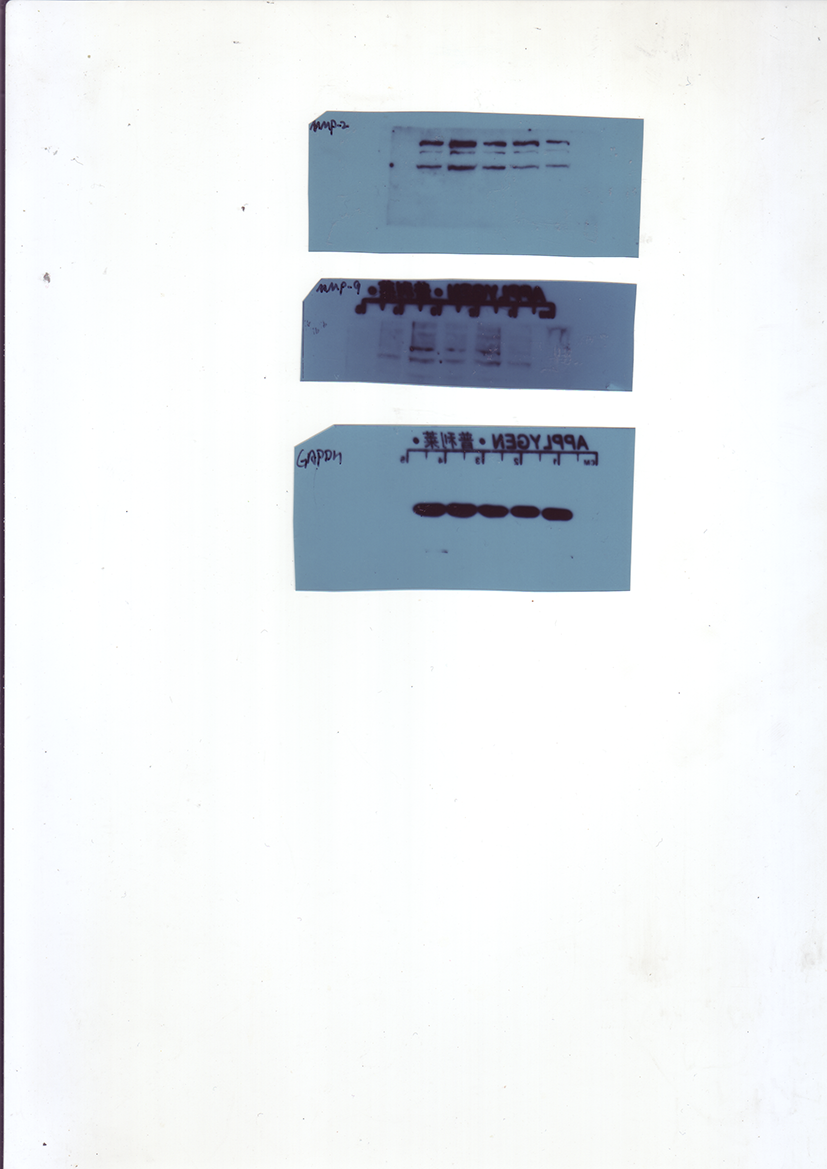

Supplement: Supplementary file 1 [file DataSheet_1.zip › Supplementary materials/Original images of Western blots(Fig 3A).tif]

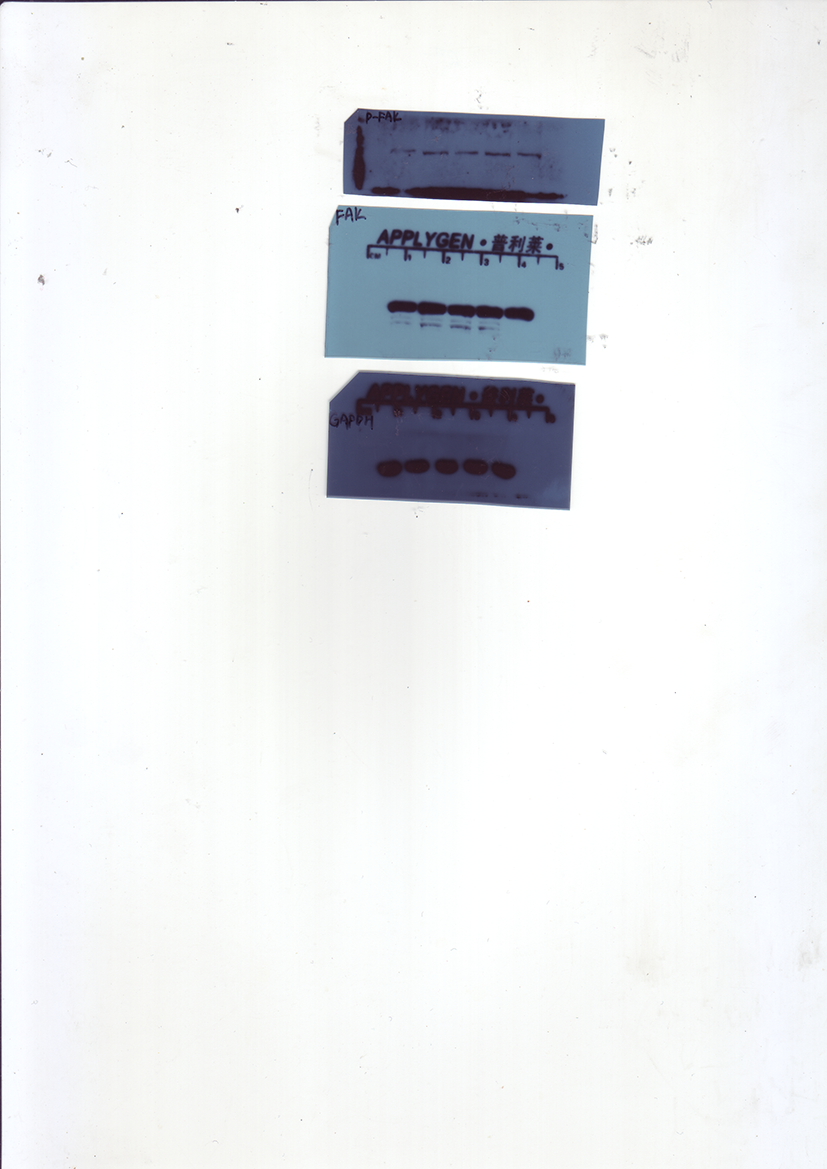

Supplement: Supplementary file 1 [file DataSheet_1.zip › Supplementary materials/Original images of Western blots(Fig 2G).tif]

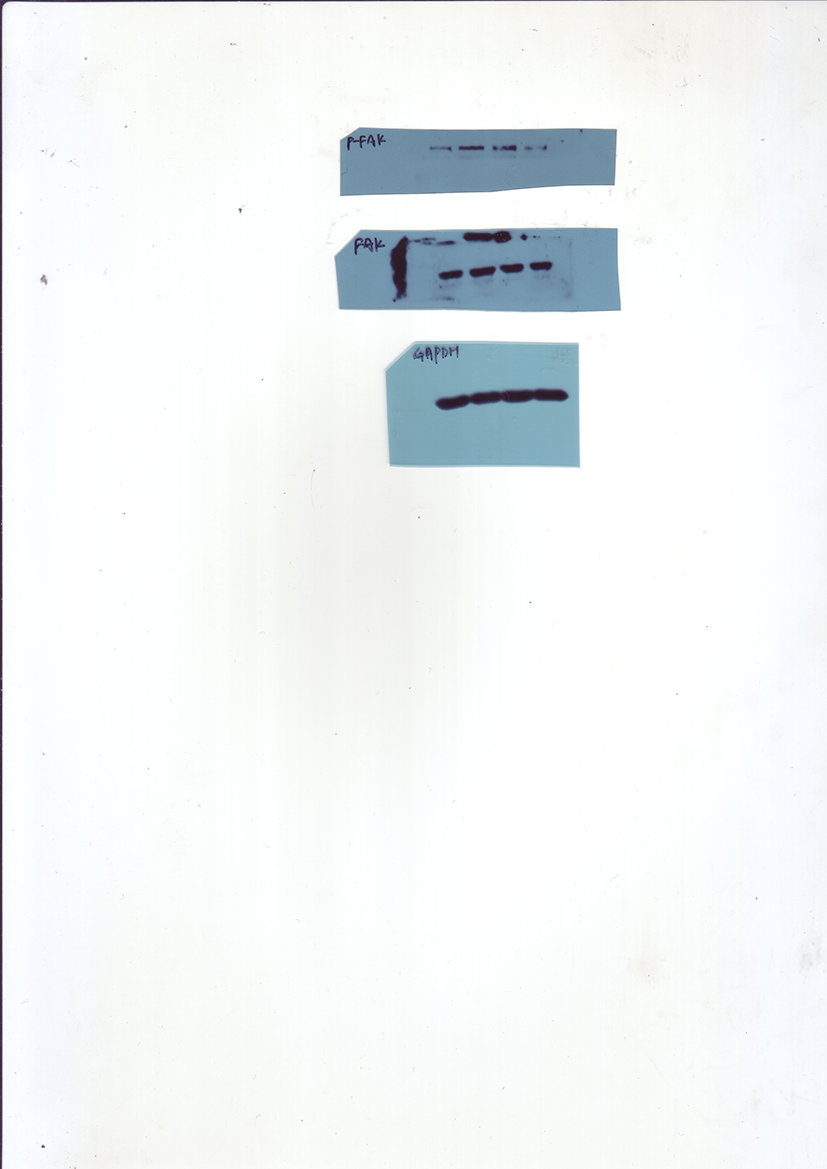

Supplement: Supplementary file 1 [file DataSheet_1.zip › Supplementary materials/Original images of Western blots(Fig 5B).tif]

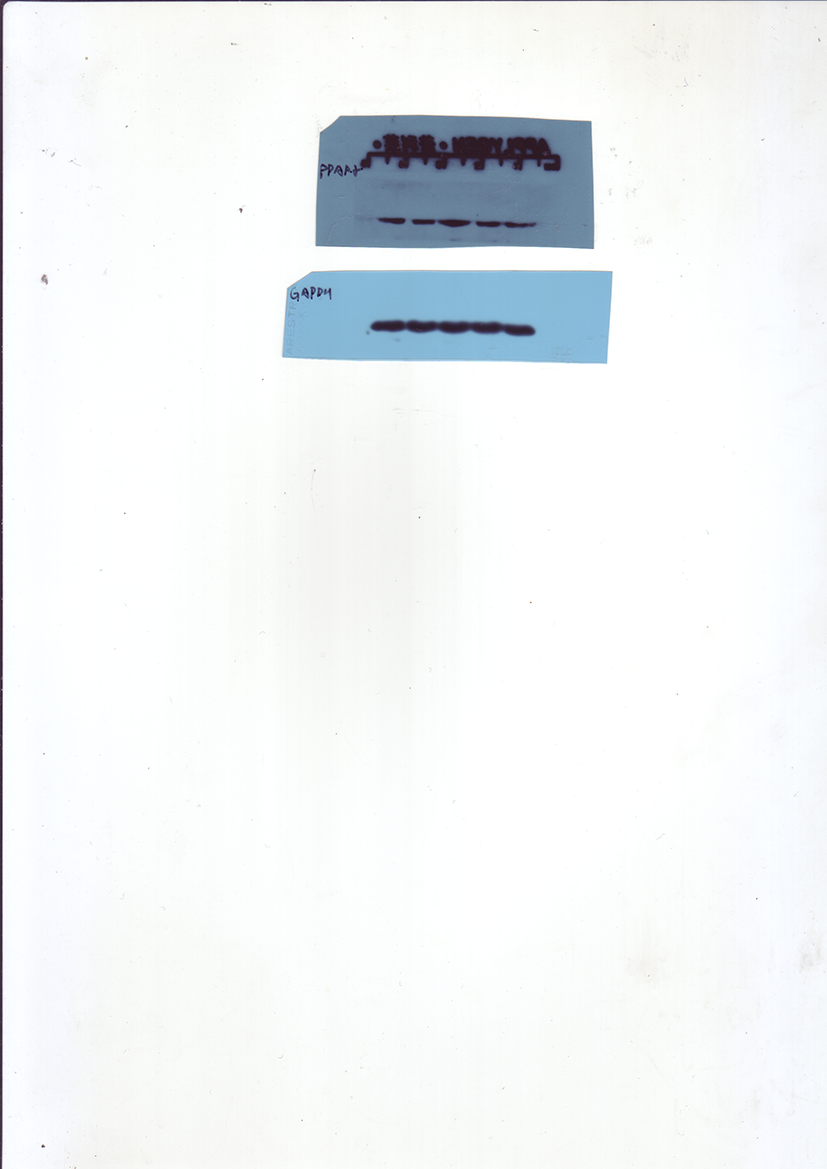

Supplement: Supplementary file 1 [file DataSheet_1.zip › Supplementary materials/Original images of Western blots(Fig 2B).tif]

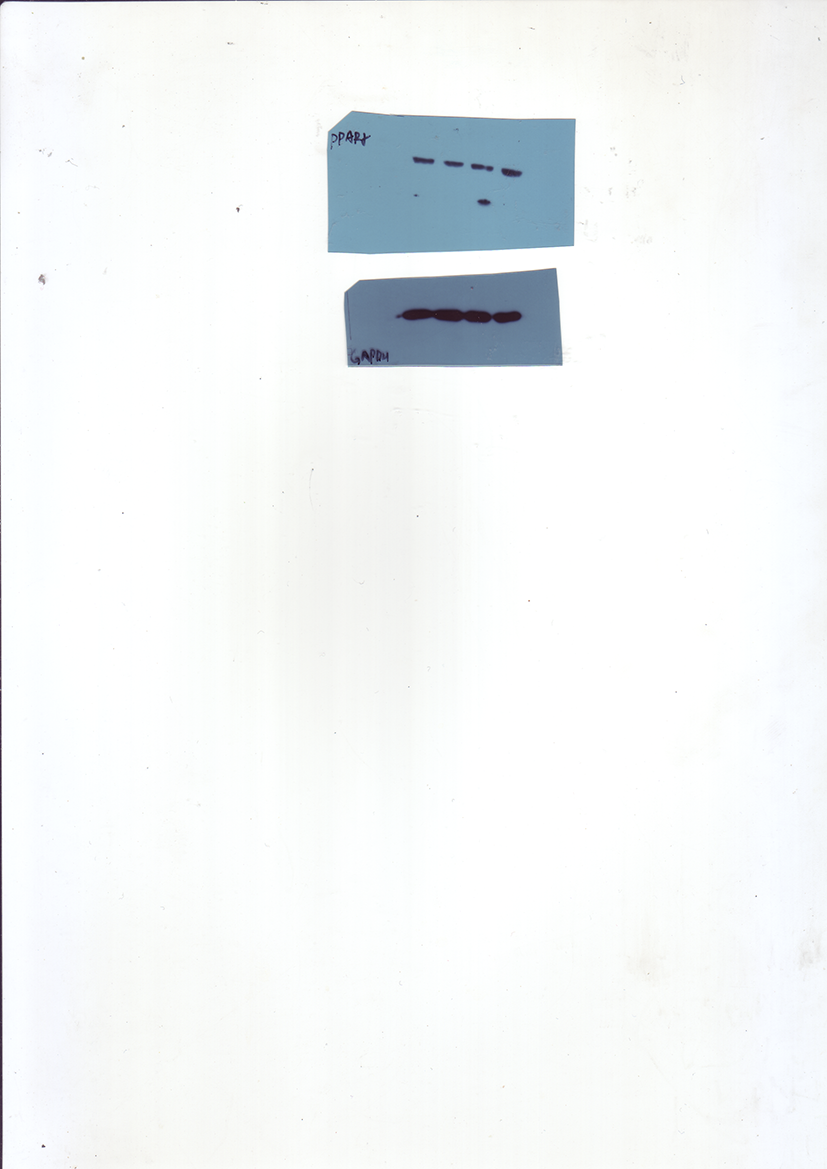

Supplement: Supplementary file 1 [file DataSheet_1.zip › Supplementary materials/Original images of Western blots(Fig 1H).tif]
